# Clostridium ramosum Bacteremia With Mesenteric Ischemia Secondary to Superior and Inferior Mesenteric Arteries Occlusion

**DOI:** 10.7759/cureus.34170

**Published:** 2023-01-24

**Authors:** Christina Padron, Mario Valdez Imbert, Abiodun M Akanmode, Ihab Jameel

**Affiliations:** 1 Internal Medicine, Columbia University College of Physicians and Surgeons, Harlem Hospital Center, New York City, USA

**Keywords:** critical care and hospital medicine, gram positive bacteremia, mesenteric ischemia, clostridium ramosum, c. ramosum

## Abstract

Clostridium ramosum, despite being a common enteric bacterium, is not commonly identified as the cause of pathologic infections in humans. It was first identified by Veillion and Zuber in 1898 from a patient with pulmonary gangrene and appendicitis. After performing an extensive literature search of major databases, only a few cases of pathologic C. ramosum infection were found in the medical literature. In this piece of work, we add to existing research by presenting a case report of an 83-year-old female who presented with abdominal pain, fever, and shortness of breath, requiring ICU admission due to mesenteric ischemia and C. ramosum bacteremia.

## Introduction

Clostridium ramosum is an anaerobic, nonmotile, endospore-forming gram-positive rod that is a typical inhabitant of the gastrointestinal tract and is often isolated from stool samples of children. This species of Clostridium tends to be misidentified due to its gram-stain variability, the lack of visualization of spores, and the atypical morphology of its colonies. In the review of current C. ramosum literature, we came across 32 cases, most of the bacteremia in adults were elderly and immunocompromised with conditions including diabetes, excessive alcohol intake, liver cirrhosis, hematologic malignancies, and organic malignancies [1]. C. ramosum has also been reported to cause osteomyelitis, septic arthritis, mastoiditis, spondylodiscitis, otitis media, pyelonephritis, septic arterial emboli, endocarditis, gas gangrene, septic pseudoarthrosis, peritoneal dialysis-related peritonitis, liver abscess, brain abscess, cerebellar abscess, lung abscess, Fournier’s gangrene, pseudomembranous colitis, infected thoracic aortic aneurysm and infection of intracranial hydatid cyst [2]. Advanced age increases the risk of clostridial infection, independent of comorbidities, which could be explained by the age-related increase of clostridial species in the normal intestinal microbiota [3].

## Case presentation

An 83-year-old woman was brought from a nursing home to the emergency department due to fever, shortness of breath, and abdominal pain for two days prior to the presentation. Her past medical history consisted of dementia, type 2 diabetes mellitus, hypertension, multi-vessel coronary artery disease (CAD) status post coronary artery bypass grafting (CABG) and stenting, chronic kidney disease stage G3b (CKDG3b), colon cancer status post colectomy, and right above knee amputation. The patient's co-morbidities were generally controlled with medications. Her home medications were Amlodipine, Aspirin, Atorvastatin, Mirtazapine, Ferrous sulfate, Ondansetron, and Insulin Glargine. In addition, she has a known allergy to lisinopril, turkey, and tomato.

**Figure 1:**
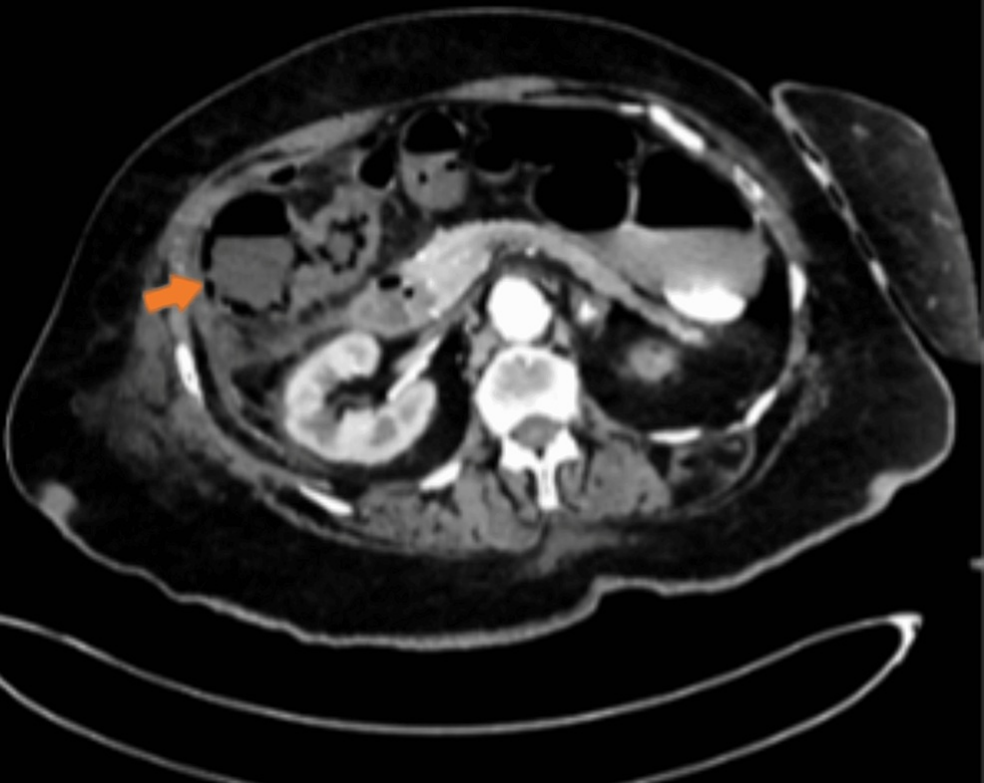
Axial CT angiogram of the abdomen and pelvis showing Pneumatosis intestinalis (orange arrow)

**Figure 2:**
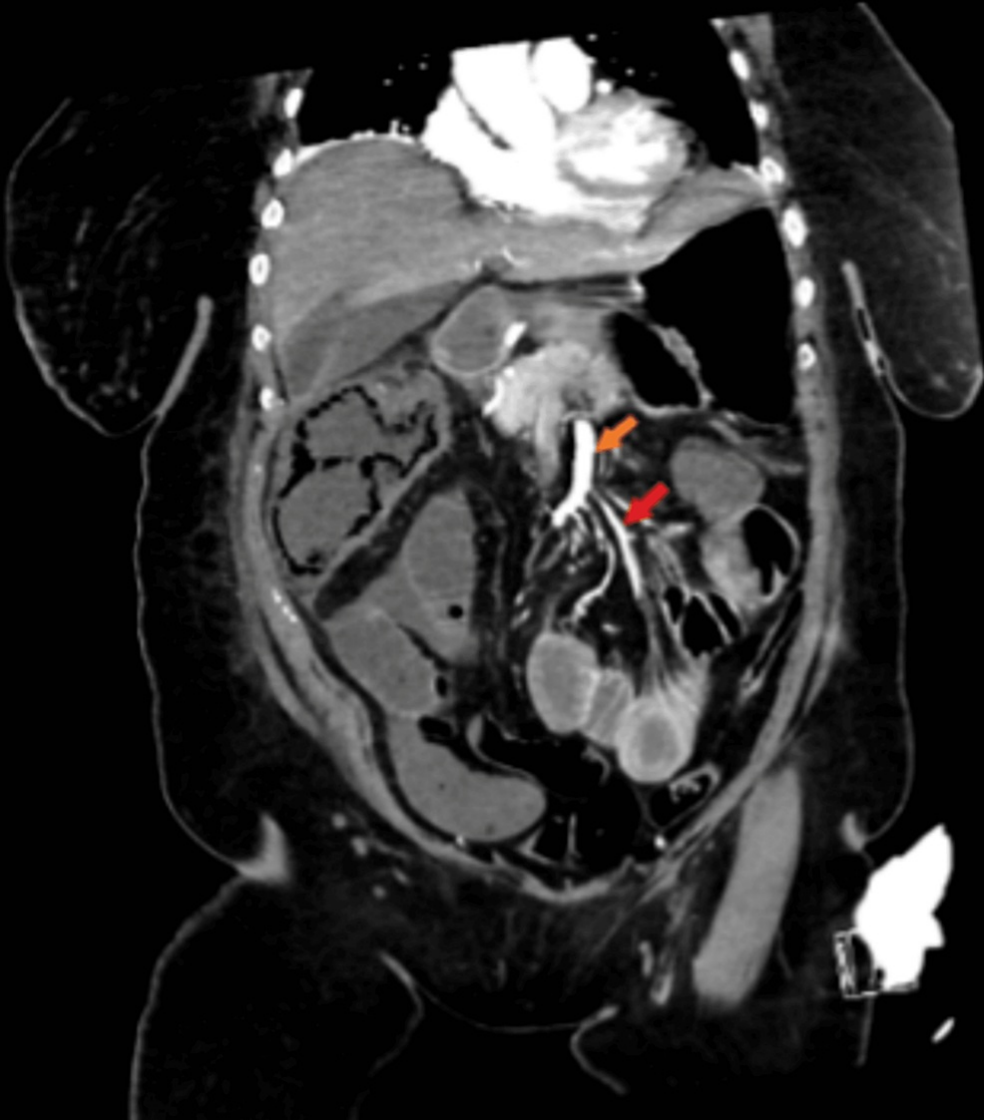
Coronal section of CT angiogram abdomen and pelvis showing descending aorta with occlusion (orange arrow), and inferior mesenteric artery (IMA) with occlusion (red arrow).

Physical examination was notable for a temperature of 101.1 F, tachycardia of 131 beats per minute, tachypnea of 27 breaths per minute, and blood pressure of 121/76 mmHg. Auscultation revealed normal heart sounds with no murmurs; diminished breath sounds on the right side. The abdomen was diffusely tender to palpation with guarding, no rebound tenderness, positive Murphy's sign and reduced bowel sounds, and digital rectal examination was unremarkable. Neurological examination was unremarkable. The calves were soft and non-tender, peripheral pulses were intact, and no pedal edema. Initial laboratory tests revealed leukocytosis of 11,550 per microliter with a predominance of neutrophils 85.6%, blood urea nitrogen 70 mg/dL (7-18 mg/dL), creatinine 4 mg/dL (0.1- 1.2 mg/dL), lactate on venous blood gas 4.3 mmol/L (0.6-1.4 mmol/L). An initial electrocardiogram (EKG) showed sinus tachycardia with premature atrial complexes. Computed tomographic (CT) abdomen and pelvis without contrast showed apparent perihepatic fluid collection, gas suspected stool in the colon, CT chest without contrast showed moderate right pleural effusion. Meanwhile, a CT angiogram abdomen and pelvis showed extensive pneumatosis-intestinalis involving the ascending colon extending to the level of the hepatic flexure (Figure 1), with moderate associated colonic wall thickening, and adjacent mesenteric/peri colonic fat stranding and edema plus fluid consistent with mesenteric ischemia, near complete occlusion of the superior mesenteric artery and inferior mesenteric artery (Figure 2), occlusive thrombus within the right common iliac artery.

Due to these findings, intravenous fluids, intravenous vancomycin, and piperacillin-tazobactam were initiated for broad-spectrum coverage, also Heparin infusion 12 units/kg/hr. Later that day, vascular surgery recommended surgical intervention in view of arterial occlusion, but the patient deferred after the benefits/risks were discussed. Preliminary blood cultures grew in anaerobic bottle gram variable rods, and metronidazole intravenous was added to the regimen for improved anaerobic coverage. On day 3 of hospitalization, final blood cultures grew C. ramosum; the identification technique used was DNA PCR assay.

Throughout this patient’s hospital stay, vancomycin was discontinued, and she completed a 10-day antibiotic course with double anaerobic coverage with Piperacillin-tazobactam and Metronidazole. The Heparin drip was stopped due to hematemesis and melena. Susceptibility came back as Metronidazole resistant and sensitive to Imipenem, and antibiotics were changed to Meropenem 2 grams every 12 hours. The patient’s symptoms subsided, abdominal pain resolved, and repeat blood cultures were negative. Revascularization was deemed unnecessary as per vascular surgery and Interventional radiology; obstruction is distal in both Superior and inferior mesenteric arteries.

## Discussion

Forrester et al. described that patients with C. ramosum fall into two demographic groups: young children with ear infections or immunocompromised adults with bacteremia [4]. In this case report, our patient was of advanced age, obese, bedridden, and diabetic making her immunocompromised and, to the best of our knowledge, the first case of acute mesenteric ischemia with severe atherosclerosis with near occlusion of the superior mesenteric artery and inferior mesenteric arteries associated with C. ramosum. The exact mechanism is not yet known, and more studies are required to answer this question. The significance of Clostridium being isolated from blood culture samples has been an ongoing debate within the microbiology and pathology community because some believe this species is non-pathogenic and should be dismissed as a contaminant. However, others believe this should never be dismissed and should be considered significant as it has established pathogenic potential as is present, mainly if it is found in both anaerobic and aerobic culture bottles, as with our patient [5-8]. They behaved differently when reviewing the literature to compare Clostridium as a contaminant in blood culture vs. cases with bacteremia.

Bacteremia patients were older, more seriously ill (sepsis or septic shock), and had a higher frequency of gastrointestinal diseases, especially colorectal tumors, than the general hospital population where blood cultures were obtained [5]. Thus, when determining a choice of antibiotic(s), the clinician should consider that C. ramosum is often mixed with Enterobacteriaceae therefore, using Metronidazole (a broad-spectrum betalactam with a beta-lactamase inhibitor) in combination with Meropenem or Piperacillin/Tazobactam is an excellent first choice selection, even though in our case C. ramosum was resistant to Metronidazole. The vascular complications of C. ramosum are yet to be established, but more and more cases are being reported. Kozaki et al. described an infected thoracic aortic aneurysm with C. ramosum from resection specimens, which is a life-threatening condition as it may lead to death from aortic dissection or rupture [9]. Also, C. ramosum infection can be fatal, in a study of 22 C. ramosum bacteremia cases, nine cases (41%) had a fatal outcome [2].

## Conclusions

In conclusion, one should not disregard a C. ramosum infection in an adult as a contaminant because bacteremia with this species has a high mortality rate. The clinical significance of treating these patients early and aggressively to eliminate infectious foci should be the priority. Moreover, correctly and rapidly identifying this species is essential in the bacteriological diagnosis of C. ramosum bacteremia.
